# Fungal Rhinosinusitis Caused by a *Curvularia* sp. Infection in a Female Sumatran Orangutan: A Case Report

**DOI:** 10.3390/pathogens11101166

**Published:** 2022-10-09

**Authors:** Richard R. E. Uwiera, Shyan Vijayasekaran, Alisa M. Wallace, David J. Reese, Audra L. Walsh, Trina C. Uwiera, Rebecca Vaughan-Higgins, Simone D. Vitali

**Affiliations:** 1 Department of Agricultural, Food & Nutritional Science, University of Alberta, Edmonton, AB T6G 2R3, Canada; 2 Otolaryngology Head and Neck Surgery, Medical School, University of Western Australia, Perth, WA 6009, Australia; 3 Taronga Western Plains Zoo, Dubbo, NSW 2830, Australia; 4 VetCT Consultants in Telemedicine Pty. Ltd., Fremantle, WA 6160, Australia; 5 Vetpath Laboratory Services, Jandakot, WA 6164, Australia; 6 Department of Surgery, University of Alberta, Edmonton, AB T6G 2R3, Canada; 7 Perth Zoo, South Perth, WA 6151, Australia; 8 Wildlife Health Australia Inc., Mosman, NSW 2088, Australia

**Keywords:** *Curvularia* sp., rhinosinusitis, endoscopic sinus surgery, orangutan

## Abstract

Mycotic nasal cavity and paranasal sinus infections in non-human primates (NHPs) are relatively uncommon diseases of the upper respiratory tract. This case study describes the clinical and pathological features as well as the diagnostic techniques and interventions applied to treat the associated disease. A 23-year-old primiparous female Sumatran orangutan residing at Perth Zoo in Western Australia developed intermittent episodes of right-sided epistaxis. An ulcerative nasal mass was identified from a diagnostic endoscopy. The mass was initially biopsied and showed the morphological characteristics of a dematiaceous fungal organism upon a histological examination. There were prominent mucosal and submucosal granulomatous infiltrates containing histocytes, giant cells, and lymphocytes admixed with fewer numbers of neutrophils and eosinophils surrounding the fungal organism. The organism was identified as *Curvularia* sp. by the fungal characteristics associated with the histopathology, culture growth, and PCR analysis. The mass was subsequently removed with endoscopic sinus surgery (ESS) and the orangutan was medically treated with itraconazole for several months. The recovery was uneventful and the orangutan returned to full health.

## 1. Introduction

An infection of the respiratory tract in non-human primates (NHPs) can be a common manifestation of disease within this group. The disease can be described by either the infectious agents such as viruses, bacteria, parasites, or fungi or by the anatomical location of the disease, i.e., the upper and lower respiratory system. Often, both the infectious agent and the location of infection are used in concert to present the most accurate description of the disease and disease process.

Although the anatomical demarcation between the upper and lower respiratory tract can vary [1], in general the upper respiratory tract (URT) in NHPs is broadly similar to humans even though orangutans lack a frontal sinus [2]. The anatomy of the URT includes all extrapulmonary structures described in humans; namely, the nasal cavity, sinuses, nasopharynx, trachea, larynx, and large bronchi [3]. In addition, various species of great apes and old-world monkeys also have well-developed laryngeal diverticula (i.e., air sacs), which are vestigial structures in humans [4]. The lower respiratory tract consists of structures not part of the URT and this can include smaller bronchi and bronchioles as well as alveoli with the lung parenchyma. The URT is a common site of infection in NHPs and, as with humans, can be associated with a wide variety of pathogens. Infections can occur in all structures of the upper and lower respiratory system, but the air sacs and nasal cavity have a noticeably high incidence of disease in NHPs [5,6,7].

Viruses and bacteria are commonly associated with respiratory system diseases in NHPs and can include a wide variety of agents [3,6,8]. Mycotic infections, on the other hand, are less common, but can cause severe disease. In NHPs, *Pneumocystis* sp. and dimorphic fungi appear to be common fungal agents of URT disease [9,10,11,12]. It seems reasonable to infer that respiratory disease caused by other primary or opportunistic fungal pathogens could also induce significant URT disease in NHPs and should be considered to be a potential cause of clinical disease [13]. This case report describes an incident of a nasal and paranasal sinus infection associated with *Curvularia,* a dematiaceous fungus in an orangutan. Although *Curvularia* sp. is noted to be an emerging pathogen in humans (causing phaeohyphomycosis as well as chronic mycotic rhinosinusitis), this fungus has not been previously documented to have an infectious etiology in NHPs [14,15]. Accordingly, to the best knowledge of the authors, this is the first reported incidence of fungal rhinosinusitis caused by *Curvularia* sp. in an NHP.

## 2. Material, Methods, and Results

### 2.1. Case Description and Anesthesia

An otherwise healthy 23-year-old primiparous female Sumatran orangutan (*Pongo abelii*) at Perth Zoo in Western Australia developed intermittent unilateral epistaxis from the right nostril over several weeks. The orangutan was housed with her daughter in a 190 m^2^ enclosure with daily outdoor access. Her diet included a daily provision of fresh produce (vegetables, leafy greens, fruit, and nuts); a custom-made primate pellet with varied protein sources, including egg, quinoa, and sardines; and locally sourced plant material that included *Ficus, Coprosma,* and banana leaves.

To investigate the cause of the epistaxis, the orangutan underwent a diagnostic endoscopic procedure. This procedure was performed under general anesthesia and, as such, the orangutan was fasted from solid food for eight hours and oral fluids for one hour prior to the surgery. The orangutan was premedicated with 1.5 mg alprazolam per os and then, after 60 min, was administered 2.3 mg/kg of 100 mg/mL tiletamine and zolazepam (Zoletil^®^, Virbac, Milperra, NSW, Australia) and 0.04 mg/kg medetomidine (Bova, Caringbah, NSW, Australia) intramuscularly using a dart delivery system (a 1.5 mL syringe with the deployed syringe set at a two bar projectile pressure) (Dan-Inject Rifle, Austin, TX, USA). The orangutan was intubated using an 8.0 mm cuffed endotracheal tube and maintained on isoflurane gas (1.5–2.0% at 2000 mL/min O_2_). The hemodynamics were preserved with an intravenous fluid therapy (Hartmann’s Solution, 10.5 mL/kg/hour). The clinical parameters were monitored; these included the heart rate, respiratory rate, and mean blood pressure, which were maintained at 70–80 beats/min, 30 breaths/min, and 50–60 mm Hg, respectively, throughout the procedure. The body temperature was maintained at approximately 37.0 °C by placing the orangutan on a heated blanket (Bair Hugger3M, North Ryde, NSW, Australia). Prior to the endoscopy, the orangutan was administered half the amount of a reversal agent to counter the medetomidine component of the induction regime and to improve the cardiovascular function (0.1 mg/kg atipamezole intramuscularly; Ilium Atipamezole^®^ Troy Laboratories, Glendenning, NSW, Australia). Following the completion of the procedure, the orangutan was administered the remaining reversal agent (atipamezole, 0.1 mg/kg intravenously). This anesthesia protocol was used for all the procedures described in this report.

The orangutan recovered uneventfully within her enclosure and within two hours post-surgery. The orangutan was then provided with 5.2 mg/kg of ibuprofen (Nurofen^®^, Reckitt Benckiser Pty Limited, Sydney, NSW, Australia) per os twice daily for three days for post-surgical analgesia and began a fluconazole 6 mg/kg per os twice daily (Sandoz, Macquarie Park, NSW, Australia) treatment for a suspected fungal infection for four weeks post-endoscopy.

### 2.2. Endoscopic Sinus Surgery with Biopsy and Tissue Histopathology

**Figure 1:**
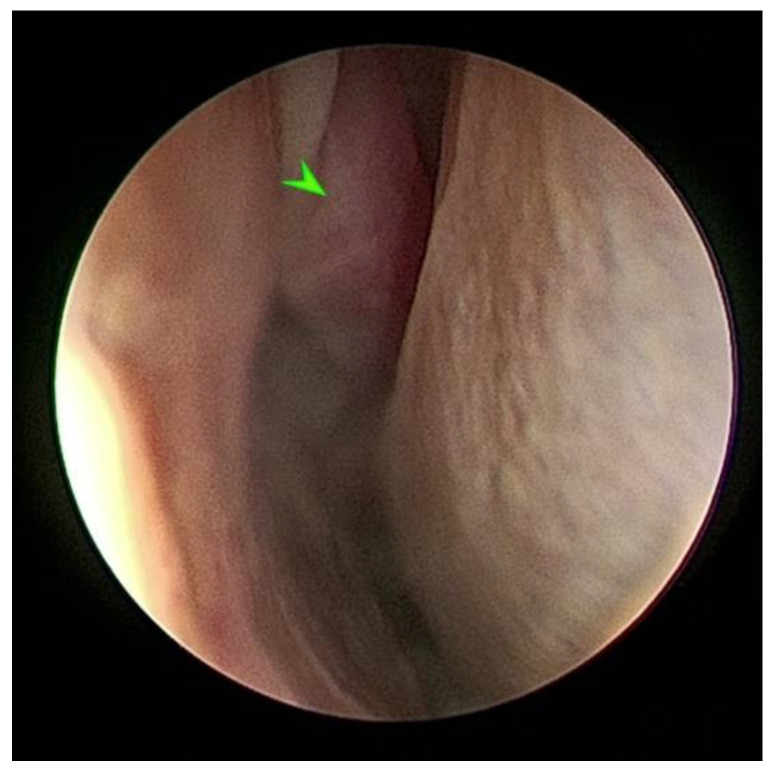
Endoscopic imaging of the nasal fungal mass. The mass caused distension of the overlying mucosa with a protrusion of the tissue into the right nasal cavity. Green arrowhead shows the fungal mass.

The diagnostic endoscopy revealed a 2.0 × 3.0 cm oblong mass that extended from the mucosal surface of the dorsal turbinate into the right nasal cavity (Figure 1).

**Figure 2:**
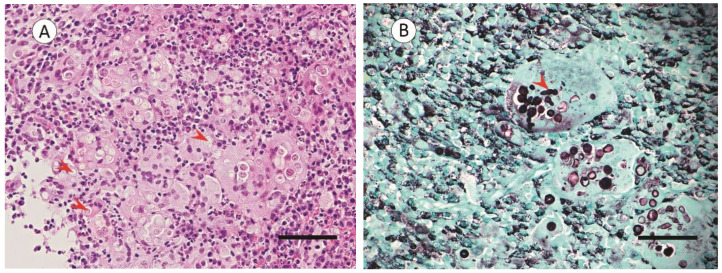
Histologic images of the biopsied nasal fungal mass. (**A**) Nasal mucosa demonstrating several thin to moderate-sized walled fungal organisms. These fungal organisms were present both intracellularly and extracellularly and were surrounded by prominent granulomatous infiltrates containing histocytes, giant cells, and lymphocytes admixed with fewer numbers of neutrophils and eosinophils. Hematoxylin–eosin stain; bar = 50 µm. (**B**) *Curvularia* sp. fungi were visualized with three transverse septa, with several fungi having terminal spores. Grocott’s methenamine silver stain; bar = 20 µm. Red arrowheads show fungi and fungal structures.

A sample of this mass was harvested and, following routine tissue processing (i.e., tissue fixation, paraffin block, and 4 µm slide preparation), was stained with a hematoxylin–eosin stain for a histological tissue assessment and Grocott’s methenamine silver stain for a fungi detection [16,17]. Bright-field microscopy (Olympus BX43 Microscope, Olympus Australia Pty Ltd., VIC, AU; Moticam^®^BTU8 Tablet Camera, Motic, San Antonio, TX, USA) was used for the histologic evaluation; this showed granulomatous inflammation within the mucosa and underlying submucosa. This was characterized by prominent infiltrates of histocytes, giant cells, and lymphoid cells admixed with fewer numbers of neutrophils and eosinophils (Figure 2A). The large clusters of inflammatory cells were associated with intracellular and extracellular fungal organisms. These fungal organisms were characterized by multiple cells separated by three transverse septa. Each cell had an obovoid appearance (Figure 2B).

### 2.3. Computerized Tomography Imaging

**Figure 3:**
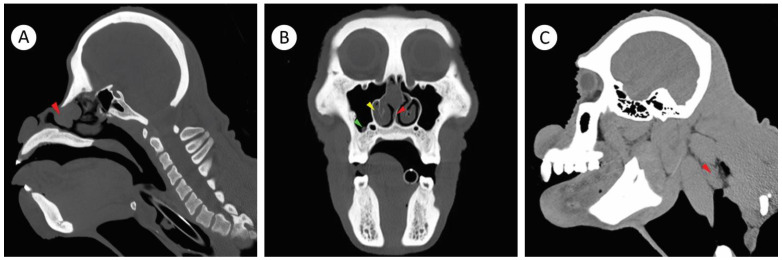
Parasagittal and transverse computerized tomography imaging of the skull and upper neck of the orangutan. (**A**) The fungal mass was present within the rostral aspect of the right nasal cavity (red arrowhead). (**B**) Leftward deviation of the nasal septum associated with the space occupying presence of the fungal mass (red arrowhead). The mucosa of the right turbinate was modestly thickened (yellow arrowhead). The fungal mass was within the right maxillary paranasal sinus over the top of the maxillary second molar tooth (green arrowhead). (**C**) The right retropharyngeal lymph node located between the buccopharyngeal and alar fascia was rounded and enlarged (red arrowhead).

Computed tomography (CT) is a valuable ancillary diagnostic test for URT disease in orangutans [18]. Therefore, three weeks following the biopsy, to investigate the extent of the infection and whether other paranasal structures were affected by the fungal granulomatous infiltrate; CT (Somatom Emotions 16 CT Scanner, Siemens, Munich, Germany) with iohexol contrast media (Omnipaque^®^, GE Health Care, Parramatta, NSW, Australia) administered under anesthesia at 523 mg/kg as an intravenous bolus - was used for the tissue enhancement. The CT imaging showed an ovoid-attenuating 3.0 cm × 1.6 cm × 1.2 cm mass within the rostral aspect of the right nasal cavity (Figure 3A). The mass caused a leftward displacement and thinning of the nasal septum as well as a decrease in the size of the left nasal cavity, resulting in a partial nasal obstruction. The mucosa of the right nasal turbinate was thickened, but there was no evidence of underlying turbinate bone erosion. The right maxillary paranasal sinus also contained a 4.2 mm × 4.2 mm × 3.5 mm mucosal mass overlying the second maxillary molar tooth (Figure 3B) and there was a conspicuous enlargement of the right retropharyngeal lymph node (Figure 3C).

### 2.4. Peripheral Blood Lymphocytes and Serum Viral Antibody Analysis and Cryptococcus Antigen Detection

The peripheral blood lymphocyte populations were quantified with flow cytometry to determine the immunocompetency of the orangutan [19], providing insight into the underlying function of the immune system and the overall health of the primate. There was mild lymphocytosis; although the cause of the elevated lymphocyte number was unknown, this may have been associated with chronic rhinosinusitis. Importantly, all lymphocyte populations, with the exception of a very slight reduction in NK cells, were within the normal limits and the orangutan was not considered to be immunodeficient (Table 1). In addition, other hematology and serum chemistry parameters were within the reference ranges and the serum antibody tests were negative for *Cercopithecine herpesvirsus-1*, *Cercopithecine herpesvirsus-2*, *Human herpesvirsus-4*, *Human T-Lymphotrophic virus-1*, *Human T-Lymphotrophic virus-2*, *Human Immunodeficiency virus-1*, *Human Immunodeficiency virus-2*, *Human Hepatovirsus A*, *Human Hepatitis B virus*, and *Human Hepacivirus C*. The orangutan had serum antibodies for *Human betaherpesvirus-5*. Finally, the orangutan was tested for the *Cryptococcus* sp. antigen, a common fungal pathogen that infects the respiratory tract of various animal species in Australia. The cryptococcus latex agglutination test (MiraVista, Indianapolis, IN, USA) was performed on the collected serum and was negative for the pathogen, suggesting that the fungal disease was not caused by *Cryptococcus* sp. (data for hematology, serum chemistry, and serum antibody titers not shown).

### 2.5. Endoscopic Sinus Surgery with Fungal Mass Excision, Fungal Identification and Medical Treatment

Five weeks after the initial biopsy, another endoscopic surgical procedure was used to excise the mass and debride the underlying infected tissue under anesthesia. This procedure involved the introduction of a 2.7 mm rigid telescope (Storz Endoscopy, Sydney, NSW, Australia) into the dorsal aspect of the right nasal cavity. This was the location of the fungal mass that extended from the right dorsal turbinate into the rostral aspect of the nasal cavity, the fungal mass was removed (Straightshot M5 Microdebrider, Medtronic, North Ryde, NSW, Australia). There were no complications or unexpected morbidities associated with the diagnostic procedure.

To determine the causative fungal agent of the rhinosinusitis, the collected fungal mass and debrided underlying mucosa were co-cultured on brain–heart infusion agar with chloramphenicol and Sabouraud dextrose agar with chloramphenicol at 35.0 °C and 30.0 °C for 3 days, respectively [23]. The abundant fungal growth (data not shown) and the presence of conidia with transverse septa and prominent terminal tapered ends on the histopathology were indicative of the genus *Curvularia* (Figure 2B). In addition, a PCR analysis of the targeted internal transcribed spacer 1 and 2 regions of the fungal ribosomal gene cluster (PCR primer ITS1; TCCGTAGGTGAACCTGCGG; IST2 GCTGCGTTCTTCATCGATGC [24]) in reference with the ISHAM-ITS database [25], and in concert with a secondary characterization of the fungus using MALDI-TOF - confirmed the fungus was *Curvularia*. The identification of *Curvularia* and the histopathological tissue findings were also well-aligned with the observations of *Curvularia* infections in humans and other mammalian species, further supporting the diagnosis of *Curvularia* as the causative agent of rhinosinusitis in the Sumatran orangutan [15,26,27,28,29].

A final diagnostic endoscopic procedure 13 weeks after the fungal mass excision was completed to ensure that there was no residual mass or inflamed or necrotic tissue within the right nasal cavity and right maxillary sinus. The endoscopy showed complete resolution of the infection. Following the diagnostic endoscopy, the fungal treatment was adjusted from fluconazole to itraconazole. Many dematiaceous fungal species have developed a significant resistance to fluconazole treatments whilst remaining relatively susceptible to an itraconazole therapy [30]. Therefore, the orangutan was administered with itraconazole 2.9 mg/kg per os twice daily (Sporanox 100 mg capsules^®^, Janssen Pharmaceutica, Macquarie Park, NSW, Australia) for 8 weeks post-endoscopy.

## 3. Discussion

In general, the anatomical structure of the URT of NHPs includes the nasal and paranasal sinus cavities, nasopharynx, larynx, air sacs (in several NHP species), trachea, and large bronchi [3,6,31]. Infections within these structures can occur at different rates and can be infected by pathogens or, in a few instances, opportunistic organisms. As an example, airsacculitis is common in many NHPs and is associated with etiologies that include viruses [32,33] and bacteria [34,35], with severe incidents of the disease being associated with infections of mixed populations of microorganisms [34]. Similar to airsacculitis, infections with multiple microorganisms can occur in the nasal cavities and paranasal sinuses of orangutans. It has been suggested that air sac infections may result from exudates draining the nasal and paranasal sinus cavities and subsequently pooling in the air sacs [35,36]. This was not determined as a source of infection for the orangutan in our case.

Naturally occurring (non-experimental) incidences of nasal and paranasal sinus fungal infections in NHPs are not well-documented. There are reported incidences of pulmonary disease caused by various fungi, but concurrent nasal or paranasal sinus infections are either not determined or investigated in clinical studies [12,37]. This is surprising, as nasal or paranasal sinus infections should readily act as a source for lung infections. Perhaps this observation reflects the ability of the fungi to colonize the respiratory mucosa or possibly our narrow understanding of the process; as there appears to be limited knowledge of fungal ecology and diversity in various NHP species [38]. In addition, this observation contrasts with the conspicuously higher incidence of primary bacterial infections within the nasal cavities and paranasal sinuses, further suggesting a limited understanding of the colonization and pathogenesis of fungi in the upper respiratory tract [6,39]. Accordingly, the identification of rhinosinusitis in endangered species—e.g., the orangutan—and its association as an opportunistic emerging fungal pathogen is of particular interest [40]. As such, and to the best knowledge of the authors, this is the first reported case of primary fungal rhinosinusitis in a Sumatran orangutan caused by *Curvularia* sp.

*Curvularia* sp. is a dematiaceous fungus that forms grey to black colonies with a velutinous mycelium [41]. These fungi have a relatively large, curved conidia and have either multiple transverse septa or distosepta as well as conidiophores with a simple, geniculate, and branched morphology [40,42]. These endophytic saprobes are present in tropical and temperate regions similar to various geographic regions of Western Australia. These fungi, although potentially pathogenic, are also mutualist to plants, liberating ions from insoluble soil mineral complexes and releasing phytohormones into the environment [43]. It is believed that these properties enable the fungi to also have a beneficial relationship with local plant species. Presently, there are more than 200 different *Curvularia* species (including varieties) registered in *Index Fungorium* [44] and, in Australia, at least 64 *Curvularia* fungi species have been recorded [45]. *Curvularia* fungi are separated into various groups based on either genotyping or the morphologic appearance within both the fungal culture and host tissues. Microscopically, individual *Curvularia* fungi are characterized by the structures of the conidiophores and the conidium. This can include the identification of the type of ornamentation on the outer conidia [46]. An identification by the morphologic appearance alone cannot differentiate the various species of *Curvularia* as there are close similarities in the morphology between individual species. As such, genotyping is becoming an important aspect of fungal identification. In our clinical study, the presence of three septa within the conidia suggested that the fungus represented one of a wide variety of *Curvularia* species, including common *Curvularia* sp. (*Curvularia lunata*), cryptic *Curvularia* sp. (*Curvularia reessii*), and others; many of these fungi are isolated in Australia [42,45]. It is important to note that although several candidate species of fungi could have caused the mycotic rhinosinusitis, the PCR analysis only verified the genus of fungi (*Curvularia*); hence, the species of the organism associated with the disease was undetermined.

*Curvularia* fungi are not only pathogenic to plants but can also be pathogenic to humans [47]. In humans, *Curvularia* sp. is considered to be an uncommon but emerging pathogen and can induce disease by either a local tissue invasion or the systemic dissemination of multiple tissues, causing significant illness in both healthy and immunocompromised individuals [48,49]. *Curvularia* sp. infections have been associated with natural and nosocomial infections. Examples of infections in people include tinea nigra [50]; superficial and deep dermatitis [49]; keratitis [41,51]; endophthalmitis [52]; periorbital ophthalmitis [15]; peritonitis [53]; stomatitis of the hard palate [14]; encephalitis of the cerebral cortex [27], midbrain [54], and brainstem [55]; and bronchiolitis and pneumonia [27,56,57]. Interestingly, *Curvularia* sp. infections of the human upper respiratory tract can induce disease within clinical observations similar to the clinical findings present within the orangutan. For example, it has been shown that *Curvularia* can establish chronic infections within the sinus and nasal cavities of humans, forming fungal granulomas that can be destructive, and can generate a prominent mixed inflammatory infiltrate rich in eosinophils, histocytes, giant cells, and necrotic cell debris [15,26,27,28]. These comparisons suggest that *Curvularia* sp. may not only cause similar lesions in NHPs and humans, but also that *Curvularia* sp. is not selective in seeding the respiratory tract structures of homidaes and can induce rhinosinusitis in both humans and great apes.

The use of endoscopy and endoscopic sinus surgery (ESS) is becoming more common for both research and to treat rhinosinusitis in NHPs [58,59,60]. An important aspect of the successful treatment of the orangutan fungal rhinosinusitis described in the study was employing ESS to both effectively biopsy and debride the *Curvularia* fungal infection. Endoscopic sinus surgery provides the surgeon with an excellent visualization of the nasal cavity and sinuses, and enables the surgeon to effectively debride the necrotic tissue and underlying bone, removing the fungal infection. Moreover, depending on the type of the open sinus surgical approach, ESS eliminates the need for an external skin incision, trephination, or the creation of osteoplastic flaps to treat fungal infections [61]. Open sinus surgical procedures not only increase the possibility of unintended complications such as delayed wound healing and nerve and bone injuries with a subsequent loss of function, but also in NHPs, carry a significant additional risk of post-surgical self-trauma and self-mutilation. With these advantages (e.g., a reduction in open sinus surgical complications), ESS is currently the primary surgical treatment for human chronic rhinosinusitis and has greatly improved post-surgery quality of life [62]. As such, it is understandable that ESS is also becoming a more accepted form of treatment of rhinosinusitis in NHPs. Although ESS provides significant advantages to diagnosis and treat nasal and sinus diseases, as with all surgical procedures there are also inherent risks. Complications identified in people include bleeding, infections, cerebral spinal fluid leaks, orbital hemorrhages, and visual impairments [62]. It is reasonable to presume that similar complications from ESS can occur in orangutans, although this has not been documented. Importantly, improved training and skills as well as an enhanced experience of the surgeon should reduce the complications associated with ESS. As such, ESS should be considered to be an effective primary treatment method for fungal rhinosinusitis for NHPs.

## 4. Conclusions

In conclusion, the cause of a right-sided epistaxis in a female Sumatran orangutan was investigated. Various diagnostic procedures, including an endoscopic biopsy, the blood lymphocyte profile, the serum viral antibody titers, the histopathology, and CT imagery were used to determine the cause of the epistaxis. It was identified, to the best knowledge of the authors, that this was the first reported incidence of a unilateral nasal and paranasal sinus infection in an orangutan caused by the dematiaceous fungus *Curvularia*. Endoscopic sinus surgery was used to successfully excise and debride the infected tissues and, with an adjunctive antifungal therapy, the procedure was curative and there was no recurrence of infection or disease.

## Figures and Tables

**Table 1:** Percentages of peripheral blood lymphocyte populations collected from a 23-year-old primiparous Sumatran female orangutan.

Lymphocyte Populations Female Sumatran Orangutan	Reference Range Human [20]	Reference Range Orangutan [21]	Reference Range Rhesus Macaques [22]
Total Lymphocytes: 92	ND	13.0–85.0	16.9–89.8
Total T cells: 69	66.0–88.1	ND	16.9–89.8
Total B cells: 21	4.7–22.5	ND	ND
CD4 T cells: 39	36.8–68.3	ND	20.3–70.2
CD8 T cells: 24	10.1–37.6	ND	0.2–5.9
NK T cells: 2	2.5–19.4	ND	ND

Values are percentages of the total numbers of lymphocytes and lymphocyte populations. Due to the limited information of lymphocyte populations in NHPs, the reference values for lymphocyte populations have been provided from the data of humans, orangutans, and rhesus macaque monkeys (for comparison). ND = not determined or not documented within the reference source.

## Data Availability

Not applicable.
